# Evaluating the impact of artificial intelligence scribes on clinical documentation in primary care: a simulation study

**DOI:** 10.1093/jamiaopen/ooag101

**Published:** 2026-07-07

**Authors:** LaShawn Murray, Emily Ha, Qianyue Wang, Isabelle Choon-Kon-Yune, Siying Luan, Vanessa Kishimoto, Onil Bhattacharyya, Payal Agarwal, Enid Montague

**Affiliations:** Department of Mechanical and Industrial Engineering, University of Toronto, Toronto, ON, M5S 3G8, Canada; Dalla Lana School of Public Health, University of Toronto, Toronto, ON, M5T 3M7, Canada; Women’s College Hospital Institute for Health System Solutions and Virtual Care, Women’s College Hospital, Toronto, ON, M5S 1B2, Canada; Department of Statistical Sciences, University of Toronto, Toronto, ON, M5G 1Z5, Canada; Women’s College Hospital Institute for Health System Solutions and Virtual Care, Women’s College Hospital, Toronto, ON, M5S 1B2, Canada; Institute for Better Health, Trillium Health Partners, Mississauga, ON, L5A 4G1, Canada; Women’s College Hospital Institute for Health System Solutions and Virtual Care, Women’s College Hospital, Toronto, ON, M5S 1B2, Canada; Women’s College Hospital Institute for Health System Solutions and Virtual Care, Women’s College Hospital, Toronto, ON, M5S 1B2, Canada; Women’s College Hospital Institute for Health System Solutions and Virtual Care, Women’s College Hospital, Toronto, ON, M5S 1B2, Canada; Institute of Health Policy, Management and Evaluation, University of Toronto, Toronto, ON, M5T 3M6, Canada; Department of Family and Community Medicine, Temerty Faculty of Medicine, University of Toronto, Toronto, ON, M5G 1V7, Canada; Women’s College Hospital Institute for Health System Solutions and Virtual Care, Women’s College Hospital, Toronto, ON, M5S 1B2, Canada; Department of Family and Community Medicine, Temerty Faculty of Medicine, University of Toronto, Toronto, ON, M5G 1V7, Canada; Department of Mechanical and Industrial Engineering, University of Toronto, Toronto, ON, M5S 3G8, Canada

**Keywords:** artificial intelligence, primary care, digital scribes, clinical documentation, electronic health record

## Abstract

**Objectives:**

To investigate the performance of artificial intelligence (AI) scribes and their impact on clinical documentation time.

**Materials and Methods:**

Artificial intelligence scribes were assessed using clinical simulations with 9 primary care physicians, each conducting 4 simulated encounters with standardized patients with and without the use of an AI scribe. Clinical simulations were audio and video recorded, and videos were coded for documentation behaviors using a designed coding scheme.

**Results:**

Clinical documentation accounts for 36.3% of the simulated clinical encounter without the use of the AI scribe, whereas primary care physicians spend 11.2% of the simulated encounter on clinical documentation when using an AI scribe. The use of AI scribes accounts for a 69.1% reduction in documentation time.

**Discussion:**

Clinical simulations offered a controlled and realistic environment to assess the impact of AI scribes on primary care physicians’ workflows, demonstrating their potential to reduce documentation time.

**Conclusion:**

Artificial intelligence scribes demonstrate potential to alleviate the burden of clinical documentation, addressing a critical factor contributing to physician burnout. Further research is needed to understand the impact of AI scribes in real-life clinical settings across complex patient scenarios.

## Background and significance

Primary care is the cornerstone of many health systems, often serving as the first point of care for patients and uniquely positioned to advance population health and reduce disparities.^1^ In many countries, primary care systems are experiencing a crisis characterized by access challenges,^2^ projected workforce shortages^3^ and provider burnout.^3^ Health care provider burnout, further intensified by the COVID-19 pandemic, continues to exacerbate this crisis impacting both providers and patients. Burnout manifests across 3 elements: depersonalization, emotional exhaustion, and reduced feelings of personal accomplishment.^4^^,^^5^ Burnout is a continued area of concern as it can reduce patient and physician satisfaction,^6^ compromise patient safety,^6^ and increase the potential for errors.^7^^,^^8^ Additionally, burnout is associated with physician turnover,^9^ compromising patients’ access to health care and the continuity of care.

Clinical documentation demands, driven by electronic health records (EHRs) adoption, have been identified as a contributor to burnout.^7^^,^^10^ Studies have demonstrated that physicians can spend up to 2 h on EHR-related administrative tasks for every hour of direct patient care.^11^ Ambient artificial intelligence (AI) scribes, also known as digital scribes, are being explored as a solution to alleviate the burden of clinical documentation and address physician burnout. Digital scribes are tools that leverage natural language processing, AI, and speech recognition to record the clinical encounter and generate a structured clinical note.^12^ By producing a structured note, AI scribes have the potential to reduce documentation time and administrative load.^13^ However, given the novelty of these tools, there is limited research on their effectiveness within primary care settings. Evaluating the performance of AI scribes within a simulated primary care context is valuable to understand the impact on physician workflow and documentation prior to real-world implementation.

### Objective

This study is part of a multiphase project that aims to investigate the impact of AI scribes within the primary care context. The objective of this current study was to assess the impact of digital scribes on clinical documentation time in a simulated clinical environment. Evaluating the performance of AI scribes through a controlled context provides critical insights into their impact on physician workflow and documentation practices prior to real-world implementation.

## Materials and methods

### Study design and setting

This study used clinical simulations to evaluate the impact of AI scribes on clinical documentation time. Clinical simulation is a method that provides for the evaluation of health care technologies prior to their adoption in real-world health care settings.^14^^,^^15^ Clinical simulations are conducted under realistic conditions and assess end users, such as health care professionals, as they use software or devices to perform realistic activities.^14^^,^^15^ These simulations can reflect observing health care professionals replicate clinical interactions with real patients or patient actors while using health care technology.^14–16^ Clinical simulations as a method enables the proactive assessment of usability for a software or device and the identification of its real-world implications on health systems, clinical workflow, patient safety, and patient outcomes.^14^^,^^15^ It further establishes the clinical validity of the results to inform real-world implementation.

**Figure 1:**
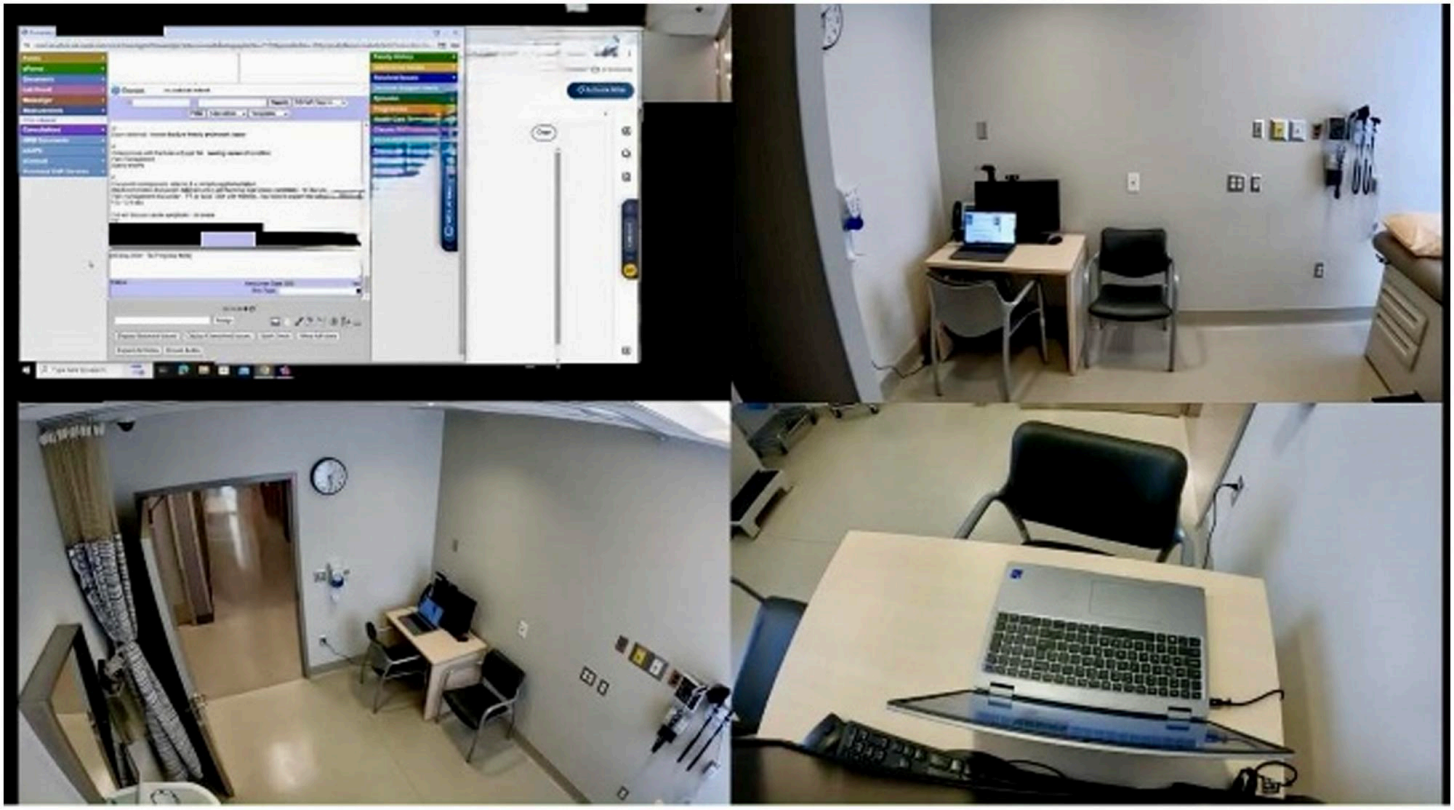
An example of the clinical simulation observation lab multichannel video orientation.

Clinical simulations were conducted at the Virtual Care Lab (VCL) at Women’s College Hospital in Toronto, Canada. The VCL is a controlled research setting that allows for the evaluation of digital health tools in a realistic clinical environment.^17^ The VCL was set up to resemble a primary care practice with an examination bed, seating area, and workstation with a laptop. The laptop was equipped with an electronic medical record (EMR) sandbox (OSCAR Pro) and various AI scribes. To observe physician behaviors, simulated encounters were audio and video recorded using 4 cameras positioned at multiple viewpoints. To capture a view of the clinic room, a GoPro camera was formatted for a wide frame angle and mounted on the ceiling. Keyboard usage was captured using a GoPro camera positioned in front of the primary care physician (PCP), angled toward the laptop keyboard. An additional GoPro camera was mounted on a tripod to capture a close-up of the provider-patient interaction. This camera had a view of the examination table, seating area and workstation. The laptop’s built-in camera was used for a view of the PCP during the interaction. The PCP’s interactions with the EMR and the AI scribe were captured through screen recording using Microsoft Teams. All videos were synchronized as a multichannel video for analysis. An example of camera layout is provided in Figure 1.

### Design of simulated encounters

Clinical simulations require end users to perform realistic tasks using health information technology. In collaboration with board-certified PCPs, the evaluation team adapted 4 standardized patient profile scenarios from the College of Family Physicians of Canada website.^18^ These patient profiles were representative of common clinical encounters observed in primary care practices, including new patient intake appointments, simple encounters and appointments addressing complex health condition(s). Two patient profiles represented complex cases and the other 2 profiles represented simple patient cases. An overview of each case is presented in Table 1. Members of the evaluation team role-played as standardized patients during the simulated encounters with a PCP.

**Table 1:** Overview of standardized patient profiles.

Name: Kennedy Jones DOB: May 23, 1967 Reason for visit: Kennedy Jones is coming in with wrist pain after a diagnosis of osteoporosis
Name: Bill Snook DOB: January 13, 1982 Reason for visit: Bill Snook is coming in with symptoms of Gastroesophageal reflux disease (GERD). They are also aware of a gambling addiction
Name: Helen Pereira DOB: March 7, 1998 Reason for visit: Helen Pereira is experiencing severe anxiety and loss of sleep. They are adamant at obtaining medication they previously used to manage their anxiety
Name: John Doe DOB: November 27, 1962 Reason for visit: John Doe is coming in after having a heart attack 3 months ago and following up after learning of their high blood pressure and type 2 diabetes

### Electronic medical record

OSCAR Pro is a web-based EMR system commonly used in primary care settings in Ontario. Participants were already familiar with the system or could easily navigate it with minimal support following a demonstration by the evaluation team. For the clinical simulations, a sandbox environment was used, which is typically employed for training or testing new digital health tools. This allowed participants to explore and interact with the EMR system and AI scribe in a controlled, risk-free setting. For PCPs, each case was to be treated as a typical visit for a new patient. Therefore, a patient chart with minimal patient history was created for each standardized patient profile in the OSCAR Pro EMR test environment. The EMR served as the interface for documentation in conditions for manual charting as well as clinical note review of an AI scribe-generated output.

### AI scribes

Up to 3 AI scribes were evaluated during the clinical simulations. The AI scribes included in this study were part of the Clinical Evaluation of Artificial Intelligence and Automation Technology to Reduce Administrative Burden in Primary Care project. This project was led by Women’s College Hospital Institute for Health System Solutions and Virtual Care and funded by the Ontario Ministry of Health through a Transfer Payment Agreement between Ontario Health and OntarioMD.^19^ Artificial intelligence scribe products were selected from the results of a competitive evaluation conducted in an earlier phase of the project. All AI scribes were web-based applications that did not integrate into OSCAR Pro EMR. Two AI scribes were browser-based applications, and the third AI scribe was a browser extension. Vendors of each product provided the evaluation team with login credentials for premium accounts to the software as well as training resources. To replicate the typical workflow, the evaluation team populated the browser-based AI scribes with appointment bookings for each standardized patient aligned with the time of the simulated encounter. This feature was not available nor required for the browser extension AI scribe.

Each AI scribe was configured to generate clinical notes in the Subjective, Objective, Assessment, Plan (SOAP) format. The SOAP format was selected as it is widely recommended for clinical documentation of patient encounters and satisfies the requirements of the College of Physicians and Surgeons of Ontario Medical Records Documentation policy.^20^

### Participants

Primary care physicians licensed to practice in Ontario were recruited via email by 4 members of the evaluation team (E.H., I.C.-K.-Y., S.L., V.K.). Recruitment began with convenience sampling, targeting participants from known networks of the evaluation team, OntarioMD, and the Ontario Ministry of Health. Following this initial phase, a provincial evaluation of AI scribes, funded by the Ontario Ministry of Health, was launched.^19^ As part of this provincial initiative, over 150 primary care providers across Ontario were given access to an AI scribe and provided informed consent to be contacted by research team members regarding participation in the simulated encounters and a post-encounter interview. Purposive sampling was employed to achieve a generally representative sample, ensuring that participants were drawn from diverse practice types and patient populations across Ontario.

### Protocol

Primary care physicians provided informed consent before conducting the clinical simulations. Primary care physicians received a patient list, indicating the patient’s name, date of birth, reason for visit and appointment time. To simulate a realistic scenario, PCPs were directed to treat each visit as a typical visit for a new patient intake where there is minimal patient history. Each session was scheduled for 90 min to allow for 4 clinical encounters, a usability survey and a postencounter interview. Two AI scribes were evaluated in each session. For 2 encounters, PCPs manually completed clinical documentation in the EMR, while the AI scribe was simultaneously running in the background (characterized as “No AI Scribe”). During the other 2 encounters, AI scribes were used for documentation. In these instances, PCPs were able to document any notes by hand to aid in recall. The simulated encounter design is presented in Table 2.

**Table 2:** Simulated encounter design.

Clinical encounter	AI scribe	Design	Patient complexity
Encounter 1	AI scribe A	No AI scribe	Simple
Encounter 2	AI scribe A	AI scribe	Simple
Encounter 3	AI scribe B	No AI scribe	Complex
Encounter 4	AI scribe B	AI scribe	Complex

Abbreviation: AI, artificial intelligence.

For each encounter, PCPs were asked to conduct a clinical interaction with a standardized patient, and complete any necessary documentation, including generating and saving a SOAP note in the patient’s chart. Standardized patients were presented in the same order. Primary care physicians were asked to complete all documentation in the clinic room prior to beginning a subsequent clinical encounter. In the “No AI Scribe” encounters, PCPs could complete manual charting simultaneously during the encounter or after the visit to mimic their usual workflow. During the AI scribe encounters, PCPs were instructed to use the AI scribe for documentation, review the scribe-generated note following the encounter, copy and paste the output into the EMR and complete any edits they deemed necessary. In either scenario, once a PCP saved the note within the EMR, the encounter was complete. As part of the broader study, following these encounters, PCPs completed a usability survey about the AI scribe and an interview was conducted; however, results from these components are not reported in this paper.

### Behavioral coding

The goal of the clinical simulations was to evaluate documentation time with and without the use of an AI scribe. The evaluation team designed a comprehensive coding scheme to categorize unique observed behaviors associated with clinical documentation. This would provide a holistic understanding of documentation tasks to better assess the efficiency of the AI scribe. The coding scheme included typing (during and after the visit), computer navigation (scrolling), interaction with the AI scribe (copying and pasting of the AI scribe-generated SOAP note) and writing. Aggregate measures included in the coding scheme were charting (typing time and writing time), documentation time and postconversation processing time (beginning at the end of the clinical encounter and including the activities from review of the SOAP note to the end of chart generation). Primary care physicians were the subject of interest for the coding scheme. Each behavior performed during the simulation was assigned a unique code to ensure consistency in analysis. A code was assigned for AI scribe generation time which represented the amount of time for the AI scribe to generate a SOAP note. A code was also assigned to document the start and end time of the clinical encounter. Supplementary Material S1 contains the clinical simulations behavioral coding scheme.

Coding was performed using Noldus Observer XT behavioral coding software. Each session was coded temporally in its entirety for the occurrence of behaviors. Behaviors were coded as state events where start and end times were coded in alignment with the coding scheme. Observations were recorded continuously in seconds. This interval was chosen based on the nature of the behaviors and the research objectives, ensuring a balance between granularity and manageability of the data.

Members of the evaluation team were assigned roles as coders. Each coder underwent extensive training to ensure reliability and accuracy in coding. Each coder was initially trained on four encounters. Training sessions included familiarization with the coding scheme, practice coding sessions using sample videos, and interrater reliability checks. Each encounter was coded by 2 researchers to establish reliability. Interobserver reliability analysis was conducted using Noldus Observer XT using the duration/sequence comparison method. This method compares whether codes overlap in between two coders, coding the same observation. The Cohen’s kappa reliability score for interobserver reliability was set to 0.90 and the percent agreement criterion of 90%. Throughout the study, reliability scores were periodically calculated. Only observations with a kappa value of 0.90 or higher were included in the data collection process. For observations where values of kappa were <0.90, the evaluation team would review the video collectively in the context of the coding scheme.

### Statistical analysis

Behavioral measures were recorded as time spent in seconds, converted into percentages of the average total encounter time at the physician level and then averaged across physicians. Observations with and without use of an AI scribe were analyzed using a 1-sided independent samples *t*-test in R Studio with statistical significance defined as *P* <.05.

### Ethical considerations

The study was formally reviewed by institutional authorities at Women’s College Hospital (WCH) and received research ethics approval from the WCH Assessment Process for Quality Improvement Projects pathway (#2023-0059).

## Results

### Participant demographics

In total, 9 PCPs participated in the simulated encounters: 4 were recruited using convenience sampling and 5 through purposive sampling. Majority of PCPs have been practicing for over 10 years (*n* = 5) and 2 for 5-10 years, and 2 for 1-4 years. The practice settings were diverse: half of PCPs worked in group- or team-based settings, 3 worked in hospital-based or academic settings, and 1 PCP had a solo practice. Notably, majority of PCPs reported serving a significant proportion of patients from historically underserved or equity-seeking populations (*n* = 6), half of which primarily provided care in a language other than English (*n* = 3). Six of the 9 PCPs had prior AI scribe experience.

### Behavioral analysis

Clinical behaviors are presented as percentages in Table 3. Percentages were used to highlight the relative differences in time spent on documentation with and without using an AI scribe, accounting for variations in provider practice styles, patient factors, and communication approaches.

**Table 3:** Clinical documentation behaviors with and without AI scribe.

Outcome	No AI scribe		AI scribe		*P*-value^a^
	Mean, s (SD)	Mean, % (SD)	Mean, s (SD)	Mean, % (SD)	
Typing					
Typing during visit	194.0 (105.5)	22.5 (12.4)	—	—	—
Typing after visit	134.6 (106.5)	13.8 (8.7)	98.6 (65.1)	11.0 (6.3)	.29
Total typing time	328.6 (90.5)	36.3 (6.6)	98.6 (65.1)	11.0 (6.3)	<.001
Computer navigation					
Scrolling time	10.2 (11.7)	1.2 (1.2)	40.3 (26.2)	4.6 (2.6)	<.001
Interaction with AI scribe					
Copy and paste time	—	—	—	—	—
Note generation time	—	—	28.7 (22.9)	3.3 (2.5)	—
Writing	—	—	1.7 (7.3)	0.2(0.9)	—
Charting	328.6 (90.5)	36.3 (6.6)	100.4 (62.7)	11.2 (6.0)	<.001
Documentation time	328.6 (90.5)	36.3 (6.6)	100.4 (62.7)	11.2 (6.0)	<.001
Postconversation processing time	155.2 (108.5)	16.2 (8.7)	209.3 (124.4)	23.2 (12.0)	.06
Total encounter time	922.8	250.1	861.0	225.1	.44

Abbreviation: AI, artificial intelligence.

a Independent samples *t*-test calculated based on proportions.

Documentation practices varied by physician. Without an AI scribe, physicians wrote their clinical notes during the clinical encounter. To assess documentation time, we calculated the sum of time spent typing, writing, and copying and pasting the AI scribe clinical note into the EMR.

On average, PCPs in this study spent 11.2% (SD = 5.99%, *P* < .001) of the total encounter on documentation when using an AI scribe as compared to 36.3% (SD = 6.59%, *P* < .001) without the use of an AI scribe. Charting accounted for time spent typing as well as any handwritten documentation. Comparable results were observed when comparing charting time with and without the use of an AI scribe as copying and pasting the clinical note into the EMR accounted for a minimal amount of time. Postconversation processing time accounts for the time from the end of the clinical encounter to completion of the SOAP note in the EMR. Primary care physicians spent, on average, 23.2% (SD = 12.04%, *P* = .06) of the encounter on postclinical documentation when using an AI scribe compared to 16.2% (SD = 8.67%, *P* = .06) without the use of an AI scribe. This additional proportion of time spent could be attributed to reviewing and editing the AI scribe-generated SOAP note prior to completing the patient chart. When using an AI scribe, editing was either conducted directly in the AI scribe or after pasting the AI scribe-generated SOAP note into the EMR. Some PCPs also completed their documentation after the clinical encounter in line with their typical workflow. Overall, the use of an AI scribe was associated with a reduction in documentation time. The total encounter time was not significantly different in scenarios with the use of an AI scribe.

## Discussion

Clinical documentation is a contributing factor of clinician burnout, an alarming phenomenon impacting the delivery of safe, high-quality, and accessible primary care. Despite being in their infancy, AI scribes are being positioned as a potential solution to the burden of clinical documentation. This study used clinical simulations to evaluate the impact of AI scribes in a primary care context. Clinical simulations provided the opportunity to assess the impact of AI scribes on provider workflow within a realistic environment, minimizing risks to patient safety while evaluating clinical validity of the AI scribes. The results indicate that manual clinical documentation accounts for, on average, 36.3% of the simulated clinical encounter. Conversely, with the use of an AI scribe, primary care providers spend 11.2% of the simulated encounter on clinical documentation. Overall, compared to encounters without an AI scribe, using an AI scribe represents a 69.1% reduction in time spent on documentation. The average encounter duration also was shorter in instances when an AI scribe was used despite additional time spent on reviewing and editing the AI scribe-generated note. However, this difference was not statistically different, requiring further analysis on additional factors influencing overall encounter time.

The findings of this study add to the growing body of evidence on the impact of AI scribes.^12^^,^^13^^,^^21–26^ An observational study evaluated the impact of ambient voice technology on primary care provider burnout and documentation burden.^25^ Frequent use of the technology was associated with reduced documentation time during and outside of scheduled hours, greater amount of information captured (increase in documentation length), reduced provider contribution to the clinical note, and lower provider disengagement.^25^ Artificial intelligence scribes were also associated with a reduction in “pajama time,”^27^ described as the time spent in the EHR outside of working hours.^13^ However, another study found that the use of ambient AI tool resulted in an increase in documentation time after hours, albeit by a small amount, yet there was a reduction in overall documentation time among providers who used the tool.^22^ Studies have also indicated overall time savings with the use of ambient AI technology.^21^^,^^24^^,^^25^ Consistent with the literature, we also found a reduction in documentation time with use of an AI scribe. While we observed variations in clinical documentation practices, this study required PCPs to complete all documentation for a patient in the clinic room prior to commencing a subsequent encounter. Future studies should investigate the impact of ambient AI scribes in clinical settings to determine the effect on a variety of physician workflows including after-hours documentation.

### Limitations

This study has several limitations. Measures for clinical documentation solely focused on charting behavior (ie, writing a medical note in SOAP format). This study did not measure time spent on other administrative tasks such as referrals, billing, and prescription orders. Future studies should include these tasks to understand the impact of AI scribes on provider workflow. Moreover, standardized patients were used in the simulated encounters for simple and complex scenarios. Although using standardized patients in these scenarios permitted the comparison of charting behaviors between providers, they may not be representative of all the diverse cases that present in primary care settings. The complexity of the simulated encounters was designed and assessed in consultation with practicing PCPs. Savings in documentation time may be attributed to additional factors within each encounter or the specific AI scribe, despite similar performance among the scribes used in the study. Future studies should compare charting using AI scribes in real world encounters prior to adoption. Additionally, primary care practitioners within this study were asked to complete all documentation in the room after the patient had left each encounter. When charting, physicians also did not have access to dot phrases or auto text within the EMR, keyboard shortcuts that save documentation time. It is not known the extent to which this is a typical practice for providers. There may be variations in charting practices that were not accounted for within this study. Research has identified that often documentation extends after scheduled work hours (“pajama time”) and accounts for an additional 1-2 h after clinical time on EHR-related tasks.^28^ Future studies should assess how AI scribes impact charting during administrative time and after-hours work. Notably, at the time of the study, very few AI scribes were integrated within common EMRs in Ontario. More commonly, the AI scribes were available as web-based applications that sit outside of the EMR. As such, the AI scribes evaluated were not integrated into the EMR. Although this was accounted for by analyzing copy and paste behaviors, as AI scribe technology evolves, it is likely that EMR integration becomes a key factor when assessing usability and effectiveness. Another limitation is that this study only reports on documentation time. Future work will report on provider perspectives regarding the AI tools used in the clinical simulations as well as the quality of the clinical note produced.

## Conclusion

This study investigated the impact of AI scribes on clinical documentation within a simulated clinical environment. The use of AI scribes was associated with a reduction in documentation time. This study builds on the existing literature that demonstrates the promising impact of ambient AI scribe technology on reducing the burden of documentation. Additional evaluations should be conducted to determine the accuracy and quality of AI-generated clinical notes, the implications of AI scribe technology on provider burnout, and the overall burden of administrative tasks, and its effectiveness in real-world settings.

## Supplementary Material

ooag101_Supplementary_Data

## Data Availability

The data underlying this article will be shared on reasonable request to the corresponding author.
