# Supplementary material for: Evaluating the impact of artificial intelligence scribes on clinical documentation in primary care: a simulation study
Source: JAMIA Open. 2026 Jul 7;9(4):ooag101. doi: 10.1093/jamiaopen/ooag101 (PMC13341013; doi:10.1093/jamiaopen/ooag101)
Supplement: ooag101_Supplementary_Data [file ooag101_supplementary_data.zip › JAMIAOpen-AI Scribes 2.0 Simmulations - Supplemental File 1.docx]

**Supplemental File 1**

**Title: Clinical Simulations Behavioral Coding Scheme**

**Manuscript:** Evaluating the impact of artificial intelligence (AI) scribes on clinical documentation in primary care: A simulation study

| **Code** | **Code Description** |
| --- | --- |
| Start of Clinical Visit | First word spoken by either primary care physician (PCP) or patient (in clinical encounter) |
| End of Clinical Visit | Last word spoken by either PCP or patient (in clinical encounter) |
| Total Visit | First to last word spoken |
| Typing | - First letter to last letter - Typing time - Breaks nested within periods of typing if less than 5s - Scrolling if less than 5s and between periods of typing (includes mousepad and arrow keys) |
| Scrolling | - Navigating the electronic medical record (EMR) - Scrolling if more than 5s between periods of typing - Scrolling after typing if typing does not resume within 5s - Navigating the AI scribe (clicking between EMR and AI scribe, minimizing/expanding windows, scrolling behavior within the EMR) |
| Writing | Paper-based notetaking |
| Reading | Reviewing the AI Scribe output |
| Scribe generation | Start: Stop button is pressed  End: Output generated |
| Copy of AI Scribe Output | Copy the output via:   - Use of copy button within AI Scribe - Control keys to select text and copy - Manual highlight and copy   *any combination of above |
| Paste of AI Scribe Output | Paste output in EMR via:   - Use of paste button/ right click menu - Control keys to paste text |
| Clinical Observation | Vocalizing findings in a physical examination |
| Post-conversation Processing Time | End of Clinical Visit (AI Scribe is stopped, PCP starts to review/edit the note) to end of chart generation (PCP saves the note in the EMR) |
